# Symptomatic Cerebral Vasospasm After Transsphenoidal Tumor Resection: Two Case Reports and Systematic Literature Review

**DOI:** 10.7759/cureus.8171

**Published:** 2020-05-17

**Authors:** Hailey C Budnick, Samuel Tomlinson, Jesse Savage, Aaron Cohen-Gadol

**Affiliations:** 1 Neurological Surgery, Indiana University, Indianapolis, USA; 2 Neurological Surgery, School of Medicine and Dentistry, University of Rochester Medical Center, Rochester, USA

**Keywords:** delayed cerebral ischemia, transsphenoidal, tumor resection, vasospasm

## Abstract

Cerebral vasospasm is a rare life-threatening complication of transsphenoidal surgery (TSS). We report our experience with two cases of symptomatic vasospasm after endoscopic TSS, alongside a systematic review of published cases. Two patients who underwent endoscopic TSS for resection of a tuberculum sella meningioma (case 1) and pituitary adenoma (case 2) developed symptomatic vasospasm. Clinical variables, including demographics, histopathology, the extent of subarachnoid hemorrhage (SAH), diabetes insipidus (DI), day of vasospasm, vasospasm symptoms, vessels involved, management, and clinical outcome, were retrospectively extracted. We subsequently reviewed published cases of symptomatic post-TSS vasospasm. Including our two cases, we identified 34 reported cases of TSS complicated by symptomatic vasospasm. Female patients accounted for 20 (58.8%) of 34 cases. The average age was 48.1 ± 12.9 years. The majority of patients exhibited postoperative SAH (70.6%). The average delay to vasospasm presentation was 8.5 ± 3.6 days. The majority of patients exhibited vasospasm in multiple vessels, typically involving the anterior circulation. Hemodynamic augmentation with hemodilution, hypertension, and hypervolemia was the most common treatment. Death occurred in six (17.6%) of 34 patients. Common deficits included residual extremity weakness (17.6%), pituitary insufficiency (8.8%), and cognitive deficits (8.8%). Symptomatic vasospasm is a rare, potentially fatal complication of TSS. The most consistent risk factor is SAH. Early diagnosis requires a high index of suspicion when confronted with intractable DI, acute mental status change, or focal deficits in the days after TSS. Morbidity and death are significant risks in patients with this complication.

## Introduction and background

Transsphenoidal surgery (TSS) is a relatively safe and very effective approach for reaching parasellar and suprasellar tumors. Cerebral vasospasm is an extremely rare, life-threatening complication of TSS that can be difficult to diagnose because of its delayed presentation and heterogeneous course [1]. The symptomatic onset of vasospasm typically occurs 5 to 10 days after surgery, mimicking the time course of vasospasm observed in the setting of aneurysmal subarachnoid hemorrhage (SAH) [2-4]. Clinically, vasospasm presents with signs and symptoms associated with ischemia in the territory of the implicated vessel(s).

Although the mechanism of post-TSS vasospasm is uncertain, postoperative SAH seems to be the most consistent risk factor. SAH has been observed in the majority of post-TSS vasospasm cases (84.6%) and is presumed to have a pathophysiological role, although vasospasm in the absence of SAH has been described [5-8]. Vasospasm is thought to be an infrequent contributor to the overall spectrum of ischemic complications after TSS, which includes direct injury to the internal carotid artery (ICA) and compression secondary to apoplexy [9]. However, the treatment of this idiosyncratic condition lacks evidence-based guidelines, and even with intensive care, the mortality rate associated with post-TSS vasospasm is approximately 30% [4,5,10].

Compared to the typical complications of pituitary tumor resection (e.g., diabetes insipidus [DI], pituitary insufficiency, cerebrospinal fluid [CSF] leak, meningitis, vision deficits), reports of symptomatic vasospasm after TSS are sparse [1,4-6,9,11-19]. As a consequence, very little is understood about the prevalence, etiology, and management of this condition. We report here our experience with two cases of symptomatic vasospasm and delayed cerebral ischemia after endoscopic TSS for resection of a tuberculum sella meningioma (case 1) and a pituitary adenoma (case 2). We also summarize our review of published cases to highlight the common presenting features, timing, clinical course, and management strategies.

## Review

Institutional miniseries

During the year of 2019, two patients who underwent endoscopic TSS for pituitary tumor resection at the Indiana University Department of Neurological Surgery developed symptomatic vasospasm within two weeks of surgery. The medical records for these patients were accessed retrospectively, and the following clinical variables were extracted from each patient’s chart: demographics (age and sex), presenting symptoms, tumor extension, histopathological diagnosis, the documented occurrence of CSF leak, identification of postoperative SAH, postoperative DI, postoperative day (POD) of vasospasm diagnosis, clinical signs and symptoms during vasospasm, diagnostic modality used to confirm vasospasm, the vessels involved, management strategy, and outcome at discharge or last follow-up.

Institutional review board/ethics committee approval and patient consent were neither required nor sought for this study.

Literature review

We searched the literature for all published cases of symptomatic vasospasm after TSS for sellar or suprasellar tumor resection. An initial set of articles was obtained by querying the Medline/PubMed database with the following search terms (similar to those used by Mansouri et al.): [“pituitary”] and [“vasospasm” or “spasm” or “delayed cerebral ischemia”] and [“resection” or “surgery” or “transsphenoidal” or “TSS” or “ETSS”] [4]. Additional articles were obtained by cross-checking references and hand searching in Medline/PubMed and Google Scholar. Articles published in a language other than English were discarded. Cases in which vasospasm was preceded by pituitary apoplexy were also excluded because of the established risk of cerebral vasospasm in patients with this disorder [4]. The literature search was performed by two independent authors (H.C.B. and S.B.T.), and inconsistencies were resolved by discussion with the senior author (A.C.G.). After screening each abstract for relevance, full-length articles were reviewed for the clinical variables listed earlier.

Statistical reporting

Descriptive statistics (means and standard deviations) are used to report group-level continuous data from the literature search. Categorical variables (e.g., presence of SAH) were reported as percentages. Variables that could not be readily categorized (e.g., symptoms during vasospasm) are presented individually.

Case 1

**Figure 1:**
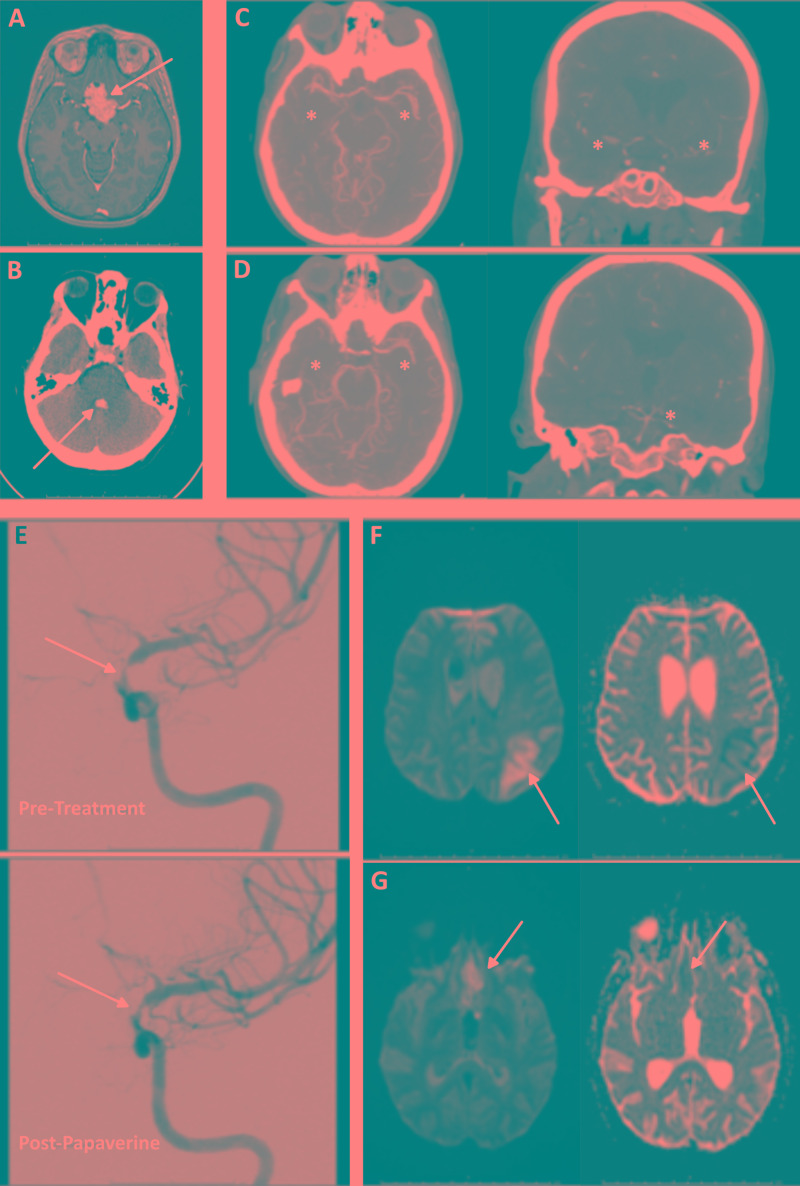
Case 1: 36-year-old woman Preoperative contrast-enhanced T1-weighted MRI reveals a large suprasellar mass with prepontine cisternal extension, encasement of the left proximal PCA, and partial encasement of bilateral supraclinoid ICA and A1 segments (arrow). (B) CT of the head shows IVH within the fourth ventricle on POD 0 (arrow). (C) CTA of the head on POD 16 suggests delayed cerebral vasospasm in the ACA and MCA territories (asterisks), worse on the left than the right side. (D) Vasospasm is also apparent in the PCA territory (asterisks), worse on the left side. (E) Digital subtraction angiography reveals severe vasospasm in the left paraclinoid ICA (arrow). (F) Left PCA infarct (DWI, then ADC) (arrows). (G) Bilateral ACA infarcts (DWI, then ADC) (arrows) CTA, CT angiography; PCA, posterior cerebral artery; ICA, internal carotid artery; IVH, intraventricular haemorrhage; ACA, anterior cerebral artery; MCA, middle cerebral artery; DWI, diffusion-weighted imaging; ADC, apparent diffusion coefficient; POD; postoperative day

A 36-year-old right-handed woman presented with a mild right homonymous hemianopsia. MRI revealed a large suprasellar mass with prepontine cisternal extension, encasement of the left proximal posterior cerebral artery (PCA), and partial encasement of the bilateral supraclinoid ICA and A1 segments (Figure 1A). She underwent endoscopic TSS, which resulted in a gross-total resection of a large tuberculum sella meningioma. A lumbar drain was used during the surgery for CSF drainage and continued postoperatively. The optic nerves were decompressed during the operation, but no significant dissection of the nerves was required. Allograft was used for closure. No obvious CSF leak or excessive bleeding was noted during the operation.

Shortly after surgery, the patient developed DI and a significant worsening of her vision. CT of her head revealed intraventricular hemorrhage (IVH) within the fourth ventricle, which prompted the initiation of dexamethasone (Figure 1B). On POD 3, she developed a nasal CSF leak, which was treated with CSF diversion via the lumbar drain. Her DI and pain remained difficult to manage, but her vision began to improve. The lumbar drain was discontinued, and she was transferred out of the intensive care unit (ICU) on POD 6. On POD 7, she was transferred back to the ICU for correction of severe hyponatremia (sodium level, 116 mEq/L) with intravenous hypertonic saline, oral salt replacement, and fludrocortisone.

On POD 13, the patient developed acute dysarthria, anxiety, right-arm apraxia, and gait instability, which spontaneously resolved over several hours. On POD 16, she had altered mental status and right hemiparesis, which prompted emergent imaging. CT of her head revealed an acute infarct of the left frontal lobe. CT angiography (CTA) of her head suggested delayed cerebral vasospasm (Figure 1C and D). Digital subtraction angiography revealed severe vasospasm in the paraclinoid left ICA, which resolved with balloon angioplasty, and mild-to-moderate left A1 and proximal left M2 vasospasm, which required super-selective intra-arterial papaverine administration (Figure 1E). Therapeutic hypertension (systolic blood pressure goal, >180 mm Hg) was initiated. Nimodipine was avoided because of the difficulty maintaining therapeutic hypertension. MRI revealed acute infarcts of the bilateral anterior cerebral artery (ACA) and left middle cerebral artery (MCA) distributions (Figures 1F and G). Transcranial Doppler (TCD) ultrasonography continued to reveal evidence for MCA vasospasm on POD 17, which prompted the initiation of vasopressors. Over the next week, she remained in the ICU and underwent daily monitoring with TCD ultrasonography. By POD 27, the patient had regained full strength and returned to her cognitive baseline. At discharge, her only lasting deficits were right-hand clumsiness and preexisting visual field deficits.

Case 2

**Figure 2:**
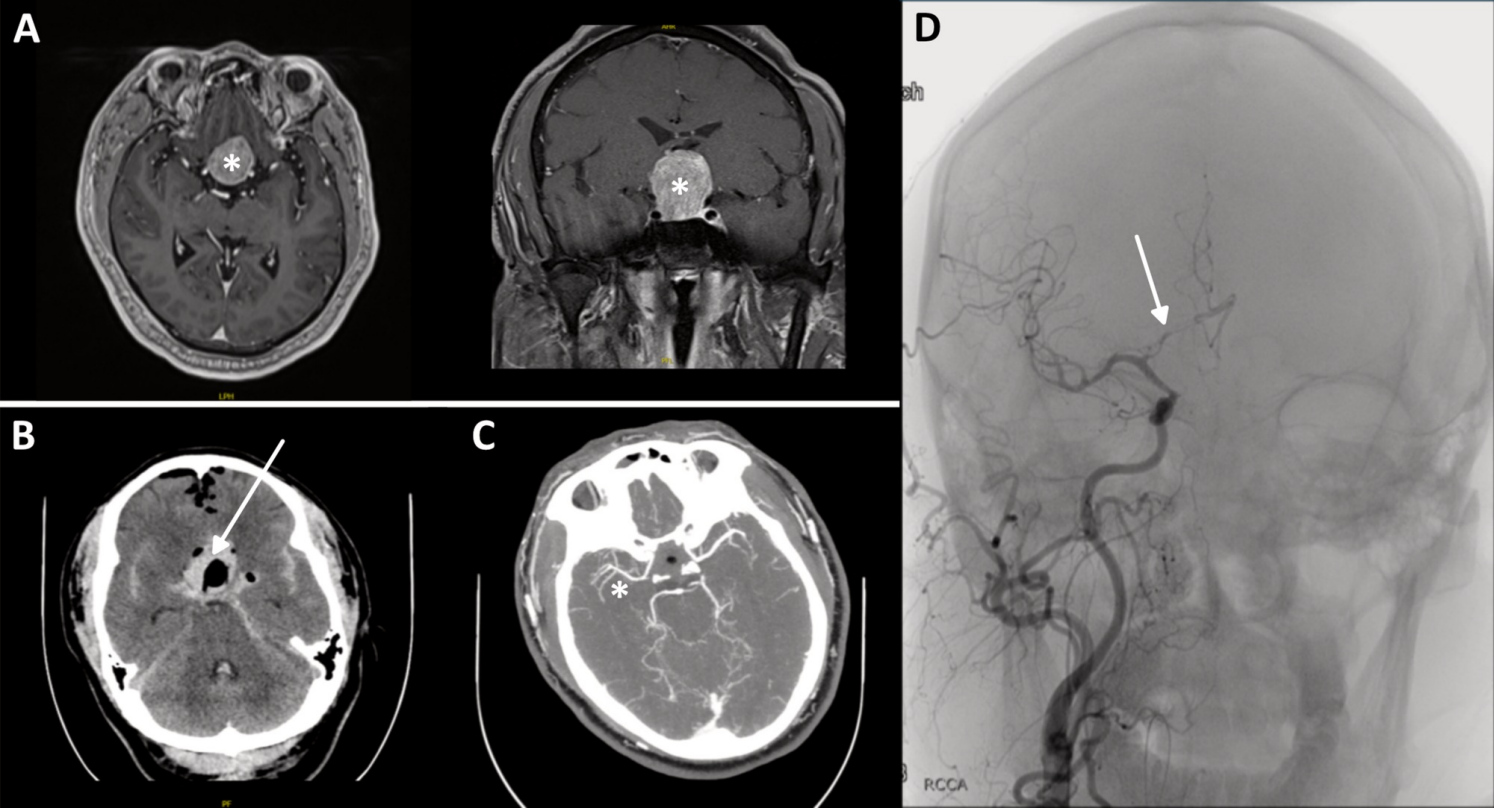
Case 2: 55-year-old man (A) Pituitary adenoma on preoperative MRI (asterisks). (B) CT of the head performed on POD 1 reveals SAH in the basal cisterns (arrow). (C) On POD 12, CTA of the head confirms vasospasm in the right MCA (asterisk). (D) Cerebral angiography performed on POD 14 reveals severe right ACA vasospasm (arrow), which was subsequently treated with intra-arterial nicardipine POD, postoperative day; CTA, CT angiography; MCA, middle cerebral artery; ACA, anterior cerebral artery

A 55-year-old man presented with a three-month history of vision loss, erectile dysfunction, and diminished libido. MRI revealed a pituitary adenoma (Figure 2A). He underwent endoscopic TSS, which was complicated by an intraoperative CSF leak. His preoperative lumbar drain was left in place after surgery for CSF diversion. The patient developed DI on POD 1, and CT of his head performed at this time revealed SAH in the basal cisterns (Figure 2B). He was started on prophylactic therapy with intravenous fluids, nimodipine, levetiracetam, and atorvastatin.

On POD 7, the patient developed altered mental status and acute lethargy. TCD ultrasonography results were consistent with bilateral MCA vasospasm. Therapeutic hypertension was initiated with a systolic blood pressure goal of >180 mm Hg. His clinical course fluctuated over the next few days in accordance with his hemodynamic status. On POD 12, CTA of his head confirmed vasospasm in the bilateral supraclinoid ICAs, MCAs, and A1 segments (Figure 2C). On POD 14, the patient was brought to the interventional radiology suite for intra-arterial nicardipine administration (Figure 2D). After endovascular treatment, no evidence of clinical or subclinical vasospasm was found, and the patient was weaned off of intravenous fluids. TCD ultrasonography results gradually normalized, and the patient was discharged from the hospital on POD 21 without any deficits. 

Literature review results

Including our two institutional cases, our literature search identified 21 articles describing 34 cases of TSS complicated by symptomatic vasospasm (Table 1). Of these cases, 13 (38.2%) overlap with the recent literature review performed by Eseonu et al., whereas the remaining cases, to our knowledge, have not been presented in this format [5]. Female patients accounted for 58.8% of the patients in these cases, and the overall mean age at the time of surgery was 48.1 ± 12.9 years (range, 19-74 years). Common presenting signs and symptoms before surgery included headache, fatigue, visual disturbance, amenorrhea, sexual dysfunction, and hypopituitarism. The most prevalent pathologies were pituitary adenoma (70.6%) and craniopharyngioma (11.8%).

**Table 1:** Reported cases of symptomatic post-TSS vasospasm: clinical characteristics ACA, anterior cerebral artery; CN, cranial nerve; CSF, cerebrospinal fluid; CTA, computed tomography angiogram; DI, diabetes insipidus; DSA, digital subtraction angiography; EBL, estimated blood loss; F, female; GOS, Glasgow Outcomes Scale; IA, intra-arterial; IVH, intraventricular hemorrhage; M, male; MRA, magnetic resonance angiography; NR, not reported; POD, postoperative day; SAH, subarachnoid hemorrhage; TCD, transcranial Doppler ultrasonography; triple H, hemodilution, hypertension, and hypervolemia; TSS, transsphenoidal surgery

Case	Age (y)	Sex	Presenting symptom(s)	Tumor type	Tumor extension	CSF leak	SAH/postop hemorrhage	Vasospasm (POD)	Diagnostic modality	Management	Outcome
Camp et al et al. [20]	33	F	Amenorrhea, galactorrhea	Pituitary adenoma	Large sellar	Yes	No	6	Cerebral angiography	Triple H, antibiotics	Death
Hyde-Rowan et al. [21]	30	F	Galactorrhea, headache, blurred vision	Pituitary adenoma	NR	No	Yes (1.4 L EBL, SAH on POD 2)	5	Cerebral angiography	Conservative	Death
Cervoni et al. [22]	51	M	NR	Pituitary adenoma	Suprasellar	NR	Yes (SAH, POD 4)	NR	TCD	Triple H, nimodipine	Complete recovery
Friedman et al. [23]	41	M	Acromegaly	Pituitary adenoma	NR	No	No	10	Cerebral angiography	Balloon angioplasty	Complete recovery
Nishioka et al. [24]	41	M	Decreased libido, vision changes	Pituitary adenoma	Suprasellar	No	Yes	12	Cerebral angiography	IA papaverine, triple H, thromboxane A2 antagonist	Stable hypopituitarism
Kasliwal et al. [14]	34	F	Amenorrhea, galactorrhea, headache, vision changes	Pituitary adenoma	Suprasellar	Yes	Yes (hematoma)	13	Cerebral angiography, TCD	Hypervolemia	Death
Popugaev et al. [25]	45	M	NR	Pituitary adenoma	Suprasellar	Yes	Yes (SAH on POD 4)	4	TCD, cerebral angiography	IV antibiotics	GOS 4
	52	F	NR	Pituitary adenoma	Suprasellar	NR	No	4	TCD	IV antibiotics	GOS 5
Zada et al. [13]	59	M	Bitemporal hemianopsia, hypogonadism	Pituitary adenoma	Suprasellar, floor of third ventricle, encasing ACAs	NR	Yes (SAH and hematoma on POD 2)	2	Cerebral angiography, CTA	IA verapamil (3×)	Short-term memory and stable visual field deficits
	66	M	Visual blurring, hypogonadism	Pituitary adenoma	Suprasellar, right frontal lobe invasion	NR	Yes (hematoma on POD 5)	8	Cerebral angiography, MRA	IA verapamil	Death
	36	F	Headache, vision change, hypopituitarism	Pituitary adenoma	Suprasellar	NR	Yes (SAH on POD 0)	9	CTA	Triple H, IA verapamil (4×)	Stable hypopituitarism
Puri et al. [1]	59	M	NR	Pituitary adenoma	Suprasellar, floor of third ventricle	No	Yes	5	Cerebral angiography	IA verapamil, triple H, nimodipine	Cognitive and visual deficits, mild weakness
	36	F	Vision changes	Pituitary adenoma	Suprasellar	No	Yes	9	CTA	IA papaverine, triple H, nimodipine	Stable hypopituitarism
	66	M	Headaches, hypogonadism	Pituitary adenoma	Suprasellar, right frontal lobe invasion	Yes	Yes	8	Cerebral angiography	Triple H, nimodipine, IA verapamil	Death
Kim et al. [11]	51	M	Vision changes	Pituitary adenoma	Suprasellar	No	Yes	9	Cerebral angiography	Triple H, nimodipine	Complete recovery
	74	M	Vision changes, headaches	Pituitary adenoma	Suprasellar	No	Yes	9	Cerebral angiography	IA papaverine, triple H, nimodipine	Complete recovery
	65	F	Vision changes	Pituitary adenoma	Suprasellar	No	Yes	7	Cerebral angiography	IA papaverine, triple H, nimodipine	Minor residual weakness
	51	F	Vision changes	Pituitary adenoma	Suprasellar	No	Yes	9	Cerebral angiography	IA papaverine, triple H, nimodipine	Complete recovery
Page et al. [8]	44	F	Headaches, vision changes, nausea	Pituitary adenoma	Suprasellar	No	No	3	TCD, CTA	Nimodipine, triple H	Tracheostomy, gastrostomy, long-term rehabilitation
Eseonu et al. [5]	43	F	Headache, fatigue, vision changes	Pituitary adenoma	Suprasellar	Yes	Yes (tumor bed hemorrhage)	12	MRA, TCD	Hypertension, euvolemia, phenylephrine, nimodipine	Complete recovery
Nash et al. [18]	48	F	NR	Craniopharyngioma	NR	No	No	11	MRI, MRA	Hypertension, nimodipine	Complete recovery
	49	F	Vision changes	Craniopharyngioma	NR	No	No	5	CTA, cerebral angiography	Hypertension, hypervolemia, balloon angioplasty, nimodipine	Mild residual dysphagia
Bierer et al. [17]	54	M	Vision changes	Meningioma	Suprasellar	Yes	No	11	MRA	Antibiotics	Subtle personality changes and impaired short-term memory
Bougaci and Paquis [15]	60	M	Vision changes, hypopituitarism	Pituitary adenoma	Suprasellar, bilateral cavernous sinuses	No	Yes (SAH and suprasellar hematoma on POD 0)	9	Cerebral angiography	Balloon angioplasty	Complete recovery
Osterhage et al. [9]	52	M	Homonymous hemianopsia	Pituitary adenoma	Suprasellar	No	Yes (SAH)	8	Cerebral angiography, TCD, CTA	IA nimodipine	Complete recovery
	55	F	Headaches, DI	Rathke's cyst	Suprasellar	No	Yes (prepontine)	13	Cerebral angiography, TCD, CTA	IA nimodipine, aspirin	Impaired left hand motor skills, CN III palsy, hypopituitarism
	42	F	Headaches, dizziness, DI	Rathke cleft cyst/ granulation tissue	NR	No	Yes (hematoma [prepontine])	12	Cerebral angiography, TCD, CTA	IA nimodipine	Complete recovery
	56	F	Hydrocephalus, chiasmal syndrome, gait and speech disturbances	Craniopharyngioma	Suprasellar	Yes	Yes (SAH, IVH)	2	Cerebral angiography, TCD, CTA	IA nimodipine	Death
Suero Molina et al. [16]	23	F	Hypopituitarism	Pituitary adenoma	Suprasellar	Yes	Yes	8	Cerebral angiography, CTA	IA and IV nimodipine, hypertension	Slight facial droop
Karimnejad et al. [2]	19	F	Headache, nausea, vomiting, fatigue, vision change	Lymphocytic hypophysitis	Suprasellar	No	No	9	MRI, MRA	Nimodipine, triple H, IA nicardipine	Global aphasia, right hemiplegia
Ricarte et al. [6]	67	F	NR	Craniopharyngioma	Suprasellar	NR	No	16	TCD, CTA	Nimodipine, hypertension	Mild paresis
Aggarwal et al. [7]	41	F	Headache, amenorrhea, vision change	Craniopharyngioma	Suprasellar	NR	No	9	DSA, CTA	IA milrinone, systemic milrinone	Complete recovery
Current study	36	F	Right-sided visual field cut	Tuberculum sella meningioma	Pre-pontine cistern	Yes	Yes	16	Cerebral angiography, CTA	Hypertension, hypervolemia, nimodipine, IA papaverine, balloon angioplasty	Slow gait and right-hand clumsiness
	55	M	Vision changes, sexual dysfunction	Pituitary adenoma	Sellar	Yes	Yes	7	TCD	IA nicardipine	Complete recovery

The majority of patients exhibited postoperative SAH or intracranial hematoma (70.6%). The timing of postoperative SAH was not consistently documented among these reports. CSF leak (29.4%) and postoperative DI (17.6%) were reported less commonly, although they were observed in both of the patients treated at our institution.

The average onset of symptomatic vasospasm was POD 8.5 ± 3.6 (range, POD 2-16). The clinical presentation of vasospasm was extremely variable across patients, with many patients exhibiting some combination of altered mental status, lethargy, and/or focal neurological deficits referable to the territory of the compromised vessel(s) (Table 1). Imaging modalities used to diagnose vasospasm included digital subtraction angiography (DSA), CTA, MRI/MRA, and TCD ultrasonography. In many patients, multiple modalities were used to confirm the diagnosis, identify the implicated vessels, and monitor for resolution or recurrence. DSA was often performed for both diagnostic and interventional purposes (see below).

The vessel(s) involved at the initial diagnosis of vasospasm was discerned in 94.1% of the cases; vasospasm in the remaining cases was described as “diffuse” [13,25]. The majority of patients exhibited vasospasm in multiple cerebral vessels, usually involving the anterior circulation (Table 2). In 40.6% of the patients, vasospasm was identified in segments of the ICA, MCA, and ACA simultaneously. The ACA was the most frequently implicated vessel, exhibiting spasm in 78.1%, with most cases involving the proximal segment. The supraclinoid segment was involved most frequently in patients with spasm of the ICA. The basilar arteries (12.5%), PCA (6.3%), and posterior communicating artery (3.1%) were rarely involved.

**Table 2:** Vessels implicated in symptomatic vasospasm ACA, anterior cerebral artery; ICA, internal carotid artery; MCA, middle cerebral artery; PCA, posterior cerebral artery; PComm, posterior communicating artery

Study	ICA	MCA	ACA	PCA	PComm	Basilar
Current study	X	X	X			
Current study	X	X	X			
Aggarwal et al. [7]	X	X	X			
Zada et al. [13]	X	X	X			
Puri et al. [1]	X	X	X			
Puri et al. [1]	X	X	X			
Puri et al. [1]	X	X	X			
Nishioka et al. [24]	X	X	X			
Kim et al. [11]	X	X	X			
Nash et al. [18]	X	X	X			
Bougaci and Paquis [15]	X	X	X			
Osterhage et al. [9]	X	X	X			
Suero Molina et al. [16]	X	X	X			
Karimnejad et al. [2]	X	X	X			
Hyde-Rowan et al. [21]	X					
Friedman et al. [23]	X					
Kasliwal et al. [14]	X					
Eseonu et al. [5]	X					
Popugaev et al. [25]		X				
Osterhage et al. [9]		X				
Cervoni et al. [22]		X	X			
Page et al. [8]		X	X			
Nash et al. [18]		X	X			
Ricarte et al. [6]		X	X			
Bierer et al. [17]		X	X			X
Osterhage et al. [9]		X	X	X		X
Zada et al. [13]			X	X		
Kim et al. [11]			X			
Kim et al. [11]			X			
Kim et al. [11]			X			
Camp et al. [20]	X				X	
Osterhage et al. [9]	X		X			X
Popugaev et al. [25]	Diffuse					
Zada et al. [13]	Diffuse					

A variety of treatment strategies were described in the literature (see Table 1). Hemodynamic augmentation with hemodilution, hypertension, and hypervolemia (triple-H therapy) was very common but not universal among the cases. In patients who underwent intra-arterial therapy, the most frequently used agents were papaverine, verapamil, nimodipine, and milrinone. It was notable that 14.7% of the patients underwent endovascular balloon angioplasty. Systemic antibiotics were used in two cases for which post-TSS meningitis (not SAH) was the presumed cause of vasospasm [25].

In terms of outcomes, death of the patient was reported in six (17.6%) cases. At the other extreme, for 41.2% of the patients, complete resolution, a Glasgow Outcome Scale score of 5, or no deficits were reported. Common deficits included residual extremity weakness (17.6%), pituitary insufficiency (8.8%), and cognitive deficits such as aphasia and memory impairment (8.8%). We found no consistency in the follow-up durations or outcome variables documented among the studies.

Discussion

Symptomatic vasospasm is a potentially deadly complication of TSS. The first reported case of angiogram-confirmed vasospasm after TSS emerged in 1980, and only a handful of cases have been reported in the years since then [20]. In this study, we add two cases recently encountered at our institution to the literature, which now includes (to the best of our knowledge) a total of 34 cases.

Prevalence of symptomatic vasospasm after TSS

Rates of post-TSS vasospasm are unknown, but existing evidence suggests that the phenomenon is extremely rare. In the most compelling study on this topic, Osterhage et al. retrospectively examined just under 2,000 consecutive microscopic TSS cases treated at their center over an eight-year period [9]. They identified symptomatic vasospasm as a postoperative complication in only four (0.2%) patients, two with a Rathke cleft cyst, one with a suprasellar craniopharyngioma, and one with a giant nonfunctioning pituitary adenoma. In all four cases, SAH preceded the onset of vasospasm. Despite the rigor and size of this retrospective analysis, the possibility that a certain number of vasospasm cases went undetected should be considered when interpreting their observed prevalence.

SAH is the major risk factor for symptomatic post-TSS vasospasm

Authors of the most recent literature review of post-TSS cerebral vasospasm evaluated 13 cases and found that SAH preceded the onset of symptomatic vasospasm in the majority (84.6%) of the patients [5]. The results of this study and others suggest a prominent role of subarachnoid blood in triggering a vasospastic response. In its own right, significant SAH is a rare complication of TSS that occurs in just 1% to 2% of cases [9,26]. There are limited data by which to ascertain how often SAH leads to the even rarer phenomenon of symptomatic vasospasm. However, Kim et al. found that 26.7% of patients who developed post-TSS SAH went on to exhibit angiogram-confirmed symptomatic vasospasm [11]. 

The pathophysiology of post-TSS vasospasm remains uncertain. Many authors have drawn mechanistic parallels to the more common occurrence of vasospasm after aneurysmal SAH. In this setting, vasospasm is frequently attributed to the effects of blood breakdown products [27]. Delayed vasospasm after open tumor resection has also been documented, especially after skull base tumor resection [28,29]. The etiology of this complication has not been elucidated, but studies have identified sphenoid wing tumor location, a diagnosis of meningioma, postoperative bacterial meningitis, and SAH as risk factors [10]. Manipulation of vessels during tumor resection is more common and more traumatic when the tumor encases the vessels, and as such, vascular encasement is a proposed risk factor for vasospasm [30,31]. Vascular tone alteration via vessel manipulation during tumor resection has also been proposed as a mechanism for postoperative vasospasm via the impairment of vasodilation [32]. Tumors have also been known to release vasogenic molecules such as calcium and lipids, which can induce vasospasm [32,33].

Sellar tumors confer a larger risk of postoperative vasospasm because of their spatial relationship to the circle of Willis. In addition to the proposed etiologies of vasospasm after tumor resection, hypothalamic injury or electrolyte disturbance can contribute to the risk of vasospasm after TSS [1,5,10,34]. Other proposed risk factors for postresection vasospasm are pituitary apoplexy, intraoperative CSF leak, basal cistern tumor extension, hyponatremia, and postoperative syndrome of inappropriate antidiuretic hormone [35]. Hypothalamic dysfunction can also induce vasospasm by triggering a sympathetic discharge and catecholamine release [36,37]. DI and the resultant electrolyte disruptions can also make a patient more susceptible to vasospasm [38]. During craniopharyngioma or dermoid cyst resection, vasospasm can be caused by cystic fluid spillage, as seen in practice [39] and in in vivo rat studies, in which cystic fluid injected into rats resulted in femoral artery vasospasm [40]. In our cases, early mild vasospasm most likely led to intractable DI because of the resultant ischemia of the pituitary stalk and adjacent neurovascular structures.

Surveillance and treatment strategies for post-TSS vasospasm vary

It has been proposed that treatment for vasospasm after TSS should be similar to that for vasospasm after aneurysmal SAH, which includes euvolemia/hypervolemia, permissive hypertension, prevention of hyponatremia, seizure prevention, and endovascular treatment, if necessary [3]. Given that this condition is relatively uncommon after tumor resection, especially transsphenoidal resection, systematic screening and treatment protocols have not been established at most institutions, including our own. Our literature review found an impressive heterogeneity of treatment strategies, including hemodynamic augmentation, systemic pharmacotherapy, intra-arterial antispasmodic medications/vasodilation, and angioplasty. There is a similar paucity of literature surrounding the issue of postoperative surveillance. Considering the potentially devastating consequences of undetected vasospasm, the index of suspicion should always remain high in the setting of acute neurologic deterioration in the face of an unchanged or nondiagnostic CT scan. Further complicating this matter is the assumption that neurologic deterioration caused by an expanding hematoma or syndrome of the inappropriate antidiuretic hormone could be treated with stricter blood pressure parameters and volume restriction, which can exacerbate vasospasm. This exacerbation can be a significant factor in poor final outcomes and should be strongly avoided.

Death is common in the setting of post-TSS vasospasm

Given the limitations of our retrospective literature review, we were unable to perform a rigorous examination of clinical outcomes over a consistent follow-up period with standardized outcome variables. However, the results of our review corroborate previous findings that suggested that death frequently occurs in the setting of post-TSS vasospasm [4,5,10]. The mortality rate observed in our analysis (17.6%) is lower than the 30.8% mortality rate documented in the review by Eseonu et al. [5]. There seems to be a temporal pattern in the reported deaths within our review; five of six cases involving death were published before 2013. Several non-mutually exclusive explanations might contribute to this pattern, including (1) increased awareness of this rare complication; (2) superior diagnostic capabilities, surveillance, and treatment options; and (3) changes in publishing trends, resulting in fewer accounts of unfavorable outcomes.

## Conclusions

Symptomatic vasospasm is a rare complication of transsphenoidal tumor resection. We evaluated a total of 34 reported cases to better understand the time course, presentation, treatment approaches, and outcomes of post-TSS vasospasm. Our results confirm that there is very limited evidence in the literature with which to make clinical decisions, leading to significant institutional or case-by-case heterogeneity. Despite intensive care, high morbidity and mortality rates are relatively common in the setting of this poorly understood complication.

## References

[1] Puri AS, Zada G, Zarzour H, Laws E, Frerichs K (2012). Cerebral vasospasm after transsphenoidal resection of pituitary macroadenomas: report of 3 cases and review of the literature. Neurosurgery.

[2] Karimnejad K, Sweeney JM, Antisdel JL (2016). Cerebral vasospasm following transsphenoidal hypophysectomy in the treatment of lymphocytic hypophysitis. J Craniofac Surg.

[3] Alzhrani G, Sivakumar W, Park MS, Taussky P, Couldwell WT (2018). Delayed complications after transsphenoidal surgery for pituitary adenomas. World Neurosurg.

[4] Mansouri A, Fallah A, Cusimano MD, Das S (2012). Vasospasm post pituitary surgery: systematic review and 3 case presentations. Can J Neurol Sci.

[5] Eseonu CI, ReFaey K, Geocadin RG, Quinones-Hinojosa A (2016). Postoperative cerebral vasospasm following transsphenoidal pituitary adenoma surgery. World Neurosurg.

[6] Ricarte IF, Funchal BF, Miranda Alves MA (2015). Symptomatic cerebral vasospasm and delayed cerebral ischemia following transsphenoidal resection of a craniopharyngioma. J Stroke Cerebrovasc Dis.

[7] Aggarwal V, Nair P, Shivhare P, Kumar K, Jayadevan E, Nair S (2019). Management of postoperative vasospasm following endoscopic endonasal surgery for craniopharyngioma: report and review of literature. Neurol India.

[8] Page PS, Kim DD, Hall GC, Koutourousiou M (2016). Cerebral vasospasms following endoscopic endonasal surgery for pituitary adenoma resection in the absence of post-operative subarachnoid hemorrhage. J Neurol Stroke.

[9] Osterhage K, Czorlich P, Burkhardt TR, Rotermund R, Grzyska U, Flitsch J (2018). Symptomatic vasospasms as a life-threatening complication after transsphenoidal surgery. World Neurosurg.

[10] Alotaibi NM, Lanzino G (2013). Cerebral vasospasm following tumor resection. J Neurointerv Surg.

[11] Kim EH, Oh MC, Kim SH (2013). Angiographically documented cerebral vasospasm following transsphenoidal surgery for pituitary tumors. Pituitary.

[12] Barrow DL, Tindall GT (1990). Loss of vision after transsphenoidal surgery. Neurosurgery.

[13] Zada G, Du R, Laws ER, Jr Jr (2011). Defining the “edge of the envelope”: patient selection in treating complex sellar-based neoplasms via transsphenoidal versus open craniotomy. J Neurosurg.

[14] Kasliwal MK, Srivastava R, Sinha S, Kale SS, Sharma BS (2008). Vasospasm after transsphenoidal pituitary surgery: a case report and review of the literature. Neurol India.

[15] Bougaci N, Paquis P (2017). Cerebral vasospasm after transsphenoidal surgery for pituitary adenoma: case report and review of the literature. Neurochirurgie.

[16] Suero Molina E, Di Somma A, Stummer W, Briganti F, Cavallo LM (2019). Clinical vasospasm after an extended endoscopic endonasal approach for recurrent pituitary adenoma: illustrative case and systematic review of the literature. World Neurosurg.

[17] Bierer J, Wolf A, Lee DH, Rotenberg BW, Duggal N (2017). Bilateral caudate nucleus infarcts: a case report of a rare complication following endoscopic resection of a tuberculum sellae meningioma. Surg Neurol Int.

[18] Nash R, Elwell V, Brew S, Powell M, Grieve JP (2016). Management strategy for treatment of vasospasm following transsphenoidal excision of craniopharyngioma. Acta Neurochir (Wien).

[19] Chong MY, Quak SM, Chong CT (2014). Cerebral ischaemia in pituitary disorders—more common than previously thought: two case reports and literature review. Pituitary.

[20] Camp PE, Paxton HD, Buchan GC, Gahbauer H (1980). Vasospasm after trans-sphenoidal hypophysectomy. Neurosurgery.

[21] Hyde-Rowan MD, Roessmann U, Brodkey JS (1983). Vasospasm following transsphenoidal tumor removal associated with the arterial changes of oral contraception. Surg Neurol.

[22] Cervoni L, Salvati M, Santoro A (1996). Vasospasm following tumor removal: report of 5 cases. Ital J Neurol Sci.

[23] Friedman JA, Meyer FB, Wetjen NM, Nichols DA (2001). Balloon angioplasty to treat vasospasm after transsphenoidal surgery. J Neurosurg.

[24] Nishioka H, Ito H, Haraoka J (2001). Cerebral vasospasm following transsphenoidal removal of a pituitary adenoma. Br J Neurosurg.

[25] Popugaev KA, Savin IA, Lubnin AU (2011). Unusual cause of cerebral vasospasm after pituitary surgery. Neurol Sci.

[26] Shu H, Tian X, Wang H, Zhang H, Zhang Q, Guo L (2015). Nonaneurysmal subarachnoid hemorrhage secondary to transsphenoidal surgery for pituitary adenomas. J Craniofac Surg.

[27] Kolias AG, Sen J, Belli A (2009). Pathogenesis of cerebral vasospasm following aneurysmal subarachnoid hemorrhage: putative mechanisms and novel approaches. J Neurosci Res.

[28] Salunke P, Sodhi HB, Aggarwal A, Ahuja CK (2013). Delayed cerebral vasospasm following surgery for craniopharyngioma. J Neurosci Rural Pract.

[29] Tao C, Wang J, Zhang Y, Qi S, Liu F, You C (2016). Predictors of acute vertebrobasilar vasospasm following tumor resection in the foramen magnum region. PLoS One.

[30] Bejjani GK, Sekhar LN, Yost AM, Bank WO, Wright DC (1999). Vasospasm after cranial base tumor resection: pathogenesis, diagnosis, and therapy. Surg Neurol.

[31] Symon L (1967). An experimental study of traumatic cerebral vascular spasm. J Neurol Neurosurg Psychiatry.

[32] Chang SD, Yap OW, Adler JR, Jr Jr (1999). Symptomatic vasospasm after resection of a suprasellar pilocytic astrocytoma: case report and possible pathogenesis. Surg Neurol.

[33] Ecker RD, Atkinson JL, Nichols DA (2003). Delayed ischemic deficit after resection of a large intracranial dermoid: case report and review of the literature. Neurosurgery.

[34] Macdonald RL, Hoffman HJ (1997). Subarachnoid hemorrhage and vasospasm following removal of craniopharyngioma. J Clin Neurosci.

[35] Douleh DG, Morone PJ, Mobley B, Fusco MR, Chambless LB (2018). Angioplasty is an effective treatment for vasospasm following pituitary apoplexy and tumor resection. Cureus.

[36] Wilkins RH (1975). Hypothalamic dysfunction and intracranial arterial spasms. Surg Neurol.

[37] Wilson JL, Feild JR (1974). The production of intracranial vascular spasm by hypothalamic extract. J Neurosurg.

[38] Smith D, Finucane F, Phillips J (2004). Abnormal regulation of thirst and vasopressin secretion following surgery for craniopharyngioma. Clin Endocrinol.

[39] Ford K, Drayer B, Osborne D, Dubois P (1981). Case report. Transient cerebral ischemia as a manifestation of ruptured intracranial dermoid cyst. J Comput Assist Tomogr.

[40] Kamal R, Jindal A, Suri A, Mahapatra AK (1999). Effect of craniopharyngioma fluid on femoral vessels of rat. Neurol Res.

